# Leptospirosis as an unusual culprit of acute pancreatitis and portal vein thrombosis in a New Yorker

**DOI:** 10.1002/ccr3.2736

**Published:** 2020-02-19

**Authors:** Iman Afzal, Rishi Thaker, Simcha Weissman, Megha Kothari

**Affiliations:** ^1^ NewYork‐Presbyterian Brooklyn Methodist Hospital affiliate of Weill Medical College of Cornell University Brooklyn New York; ^2^ Hackensack University‐Palisades Medical Center North Bergen New Jersey

**Keywords:** leptospirosis, pancreatitis, portal vein thrombosis, urban

## Abstract

Leptospirosis often takes clinicians by surprise when presenting in urban locations with unusual manifestations. This delays diagnosis and treatment which increases mortality rate. Our case illustrates the importance of taking into account the socioeconomic backgrounds, environmental exposures, and clinical presentations of patients to create a good differential diagnosis.

## Graphical abstract

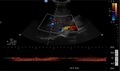

## 1 INTRODUCTION

Leptospirosis is common in tropical regions. Weil's disease, a triad of hemorrhage, jaundice, and kidney injury are perhaps the most common manifestation of leptospirosis. We present a case of Weil's disease accompanied by a rare complication of acute pancreatitis and portal vein thrombosis in a 61‐year‐old homeless New Yorker.

Leptospirosis is a zoonotic organism that is known to cause life‐threatening complications such as pulmonary hemorrhage and acute respiratory distress syndrome. Based on a systematic review of published mortality and morbidity studies, it is estimated the global burden of leptospirosis is 1.03 million cases each year.^1^ While disease manifestation may be mild or asymptomatic, severe disease can present with multiorgan dysfunction including liver failure, renal failure, pulmonary hemorrhage, vascular damage, and muscle lesions.^2^ One of the classic manifestations of leptospirosis is Weil's disease—the combination of hemorrhagic events, jaundice, and acute renal failure—first observed by Adolf Weil in 1886.^3^ We present a case of a 61‐year‐old homeless New Yorker complaining of abdominal pain who was found to have acute pancreatitis, myositis, and portal vein thrombosis due to leptospirosis in addition to renal failure and hepatic dysfunction. Our case emphasizes the importance of an early diagnosis that could be missed due to an unusual manifestation and geographical location of a disease process.

## 2 CASE REPORT

A 61‐year‐old homeless African American male with a past medical history of hypertension presented with nausea, vomiting, and abdominal pain over the last 1 week. The patient presented to a different institution 6 days prior to admission with similar complaints but left against medical advice without workup. Upon arrival to our facility, the patient's clinical status had deteriorated: He complained of intractable nausea, vomiting, fatigue, headache, photophobia, anuria, and myalgia. Patient denied any cough, fevers, chills, or night sweats. Social history included regular cocaine use. Patient denied any tobacco, alcohol, or intravenous drug use. Patient had not seen a physician since he left Rikers Island 1 year ago. While incarcerated, he denied any history of tuberculosis, HIV, hepatitis, or any other chronic diseases. He denied any recent travel history.

On physical examination, he was afebrile, with blood pressure of 177/65 mm Hg, heart rate of 77 beats per minute, respiratory rate of 16 breaths per minute, and oxygen saturation of 100% on room air. Scleral icterus, conjunctival suffusion, jaundice of the soft palate, and supple neck were noted on head and neck examination. Cardiopulmonary examination demonstrated a heaving, laterally displaced point of maximum impulse but regular rate and rhythm, normal S1, S2, no S3, or S4. On abdominal examination, patient endorsed epigastric tenderness, but demonstrated no guarding or rigidity, had normal bowel sounds and rectal examination and negative Murphy's sign.

**Figure 1:**
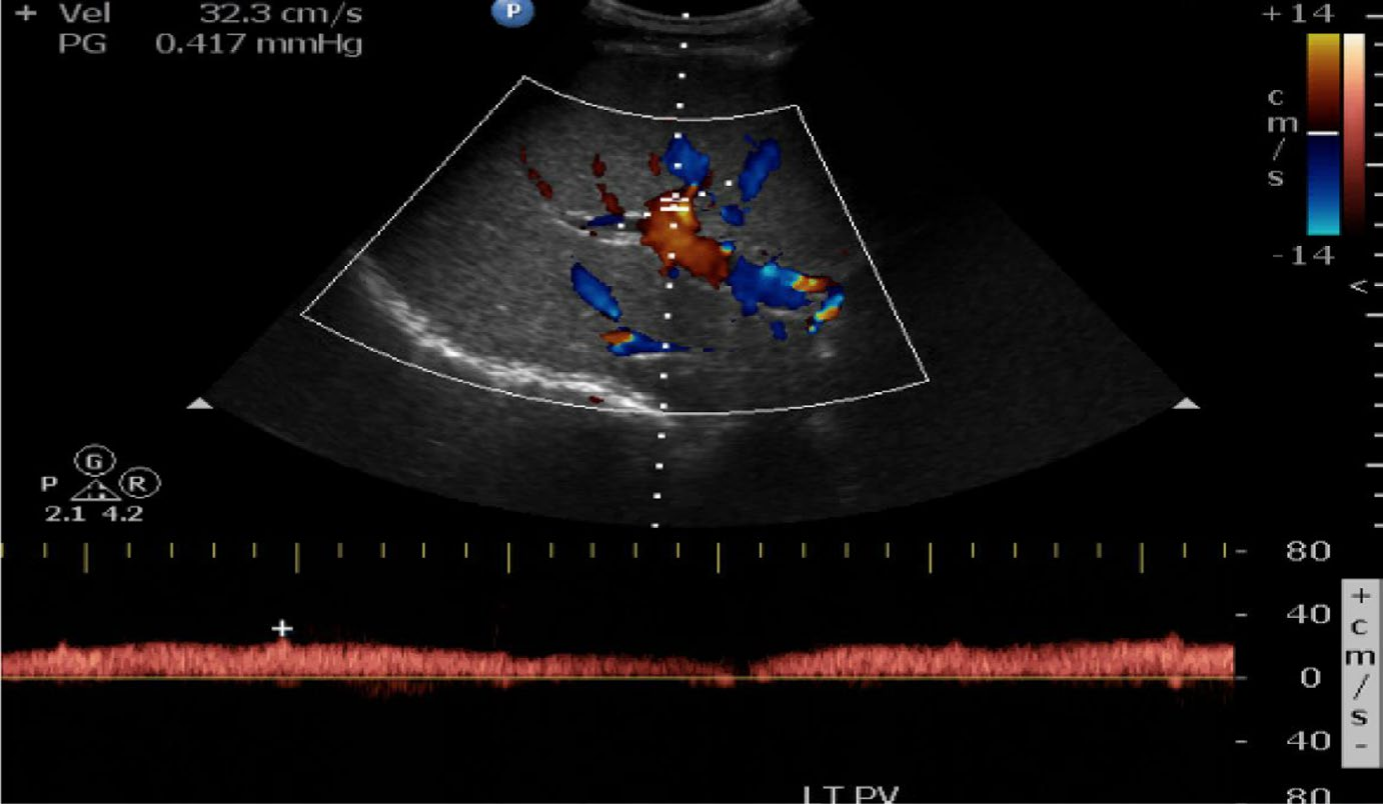
Normal directional flow and maximal flow velocity was seen in the left portal vein

**Figure 2:**
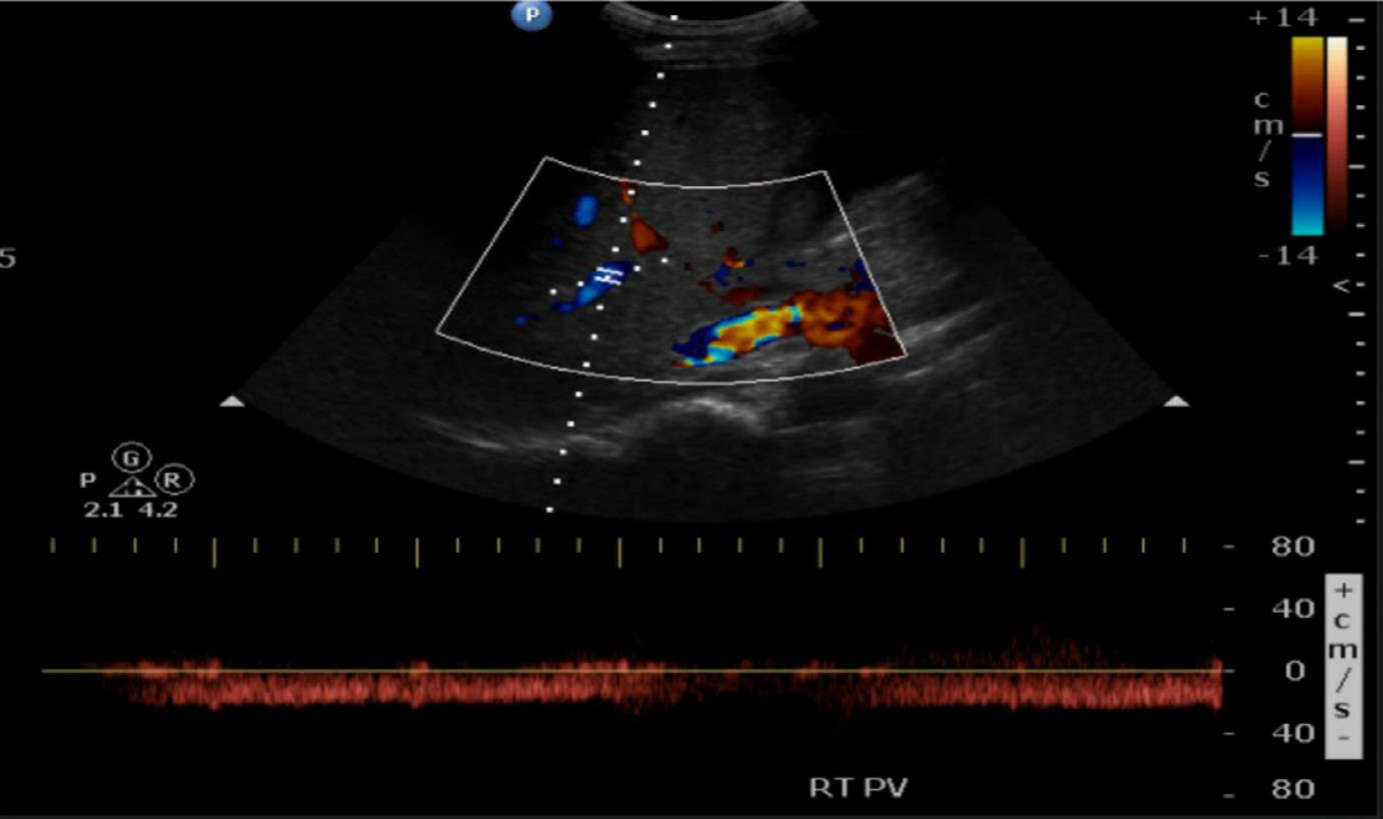
The isolated hepatofugal flow in the right portal vein is suspect for portal vein thrombosis

Initial admission laboratory values are compared to previous admission laboratory values on Table 1. Laboratories revealed a lipase level greater than three times the upper limit, mixed pattern of liver injury, thrombocytopenia, and renal failure. Urinalysis demonstrated hematuria, pyuria, small leukocyte esterase, and some hyaline casts. Initial differential diagnoses included hemolytic uremic syndrome, and thrombotic thrombocytopenic purpura given patient had low platelet counts, renal failure, weakness, abdominal pain, signs of uremia, and mild diarrhea. Blood smear showed decreased platelets counts with no schistocytes. ADAMTS13 level was normal. Stool workup was negative for infection including *Escherichia coli*, *Shigella*, and *Salmonella*. Heparin‐induced thrombocytopenia was also suspected given recent hospitalization and testing for heparin‐induced platelet antibody came back negative. Laboratory studies for underlying infections such as Cytomegalovirus (CMV), Epstein‐Barr virus (EBV), Human immunodeficiency virus (HIV) were negative. Workup for glomerulonephritis and other autoimmune conditions that involved the kidneys included antinuclear antibody, antidouble‐stranded DNA antibody, perinuclear antineutrophil cytoplasmic antibody, cytoplasmic antineutrophil cytoplasmic antibody, and complement level testing which did not show significant results. Given signs of liver dysfunction, workup for autoimmune and chronic liver diseases as well as viral causes of liver injury was investigated. This included hepatitis panel, QuantiFERON, CMV, EBV, HIV, alpha‐1 antitrypsin level, and ceruloplasmin level which also did not show substantial results. Elevated lipase levels and abdominal pain lead to the suspicion of pancreatitis. Further investigation of possible causes of pancreatitis included testing for autoimmune pancreatitis and urine toxicology screening which were both negative. Blood ethanol level was undetectable. Computed tomography of abdomen and pelvis was unremarkable except for subtle sludge vs gallstones in the gallbladder. Magnetic resonance cholangiopancreatography was unremarkable. Right upper quadrant ultrasound demonstrated isolated hepatofugal flow in the right portal vein suspicious for a portal vein thrombus (Figure 1) as opposed to the left portal vein that had normal directional flow (Figure 2). Renal function continued to worsen each day indicating a need for dialysis, which the patient refused. Treatment included starting with empiric intravenous ceftriaxone given significantly elevated procalcitonin levels, avoidance of nephrotoxic medications, pain control, and aggressive fluid hydration with lactated ringer for acute pancreatitis. Glascow Imrie score was 3, which indicated a high risk for severe pancreatitis. There was rapid improvement of the patient's abdominal pain.

**Table 1:** Comparison of previous admission values vs current admission values

Laboratory test	Previous admission	Current admission	Reference values
WBC, K/mL	13	10.1	4.0‐10.3
Hemoglobin, g/dL	13	14.6	12.5‐16.9
Hematocrit, %	39	43.1	38.3‐48.5
Mean corpuscular volume, fL	97	95.4	79.5‐98.0
RDW %	13.5	15	11.9‐15.0
Platelets, K/mL	128	89	117‐361
Glucose, mg/dL	127	76	55‐100
BUN, mg/dL	21	169	7.0‐18
Creatinine, mg/dL	1.4	10.4	0.67‐1.17
Sodium, mmol/L	141	139	136‐145
Potassium, mmol/L	4.0	4.7	3.5‐5.0
Chloride, mmol/L	103	96	100‐108
Bicarbonate, mmol/L	30	23	21‐32
Calcium, mg/dL	8.3	7.6	8.5‐10.1
Albumin, g/dL	2.3	2.2	3.4‐5.0
Total protein, g/dL	4.9	5.8	6.4‐8.2
Direct bilirubin, mg/dL	0.3	13.3	0‐0.2
Total bilirubin, mg/dL	1.1	16.1	0.2‐1.0
Alkaline phosphatase, U/L	44	105	53‐128
Alanine aminotransferase, U/L	4.9	109	8.0‐62.0
Aspartate aminotransferase, U/L	0.3	117	15‐37
Lipase, U/L	1.1	1226	73‐393
Creatinine kinase, U/L	44	625	35‐232
Magnesium, mg/dL	2.2	3.5	1.8‐2.4
Phosphorus, mg/dL	4.3	8.7	2.5‐4.9
Procalcitonin, ng/mL	N/A	136	0.00‐0.05

Given the social history of homelessness in addition to rapid progression of renal failure, liver failure along with thrombocytopenia, it was strongly suspected the patient might have a zoonotic infection. Leptospirosis polymerase chain reaction ultimately came back positive. The patient was treated with a course of doxycycline on day 2 along with symptomatic care. Over the course of a few days, the patient improved clinically, and laboratory values depicted a gradual improvement. Serum creatinine began to trend down slowly by day 3 with fifty percent reduction in value by day 5. Anticoagulation for portal vein thrombosis was initiated with weight‐based intravenous heparin on day 3 after thrombocytopenia began to improve. Patient was transitioned to apixaban indefinitely near the end of the hospital course. Subsequently, he was discharged with recommended outpatient follow‐up. Patient was lost to follow‐up.

## 3 DISCUSSION

Leptospirosis is a zoonotic organism with humans as accidental hosts. While a variety of animals act as reservoirs and shed the bacteria in their urine, the most implicated species is the brown rat (*Rattus norvegicus*).^4^ Exposure of mucous membranes to animal urine through contaminated water, mud, or direct exposure results in infection. Humans can remain asymptomatic and disseminate Leptospira into the environment for up to 1 year.^2^ Leptospirosis is common to an array of tropical climates but also poses a risk to low socioeconomic populations in city areas.^5^, ^6^, ^7^

Among symptomatic patients, one study suggests that the mortality rate for untreated leptospirosis ranges between 0% and 40% (median 2.2%).^8^ However, the mortality rate for Weil's disease exceeds 10% and the mortality rate for spontaneous pulmonary hemorrhage exceeds 50%.^8^, ^9^ Mortality risk significantly increases for patients with age >60 years, jaundice, and renal failure, while anicteric patients have remarkably low mortality.^8^ Our patient had each of these characteristics associated with high mortality, but also presented with portal vein thrombosis, myositis, and acute pancreatitis.

Characteristically, Weil's disease includes renal failure, hepatic dysfunction, and acute hemorrhage. Since leptospirosis initially colonizes the proximal tubule and disseminates via urinary shedding, the pathogenesis of renal failure is self‐evident.^10^ In hepatic dysfunction, proteases and collagenases produced by leptospires disrupt the hepatic architecture.^11^ Liver and kidney samples harvested from patients within 6‐12 hours of death from leptospirosis demonstrate the loss of E‐cadherin from the cell membrane by immunohistochemical staining, indicating a likely culprit in hepatocellular dysfunction and loss of hepatic architecture.^12^

Bleeding diathesis in leptospirosis ranges from conjunctival suffusion and petechiae, spontaneous pulmonary hemorrhage, gastrointestinal bleed, and hematuria.^13^ Typically leptospirosis is known to produce thrombocytopenia, platelet dysfunction, and multifactorial coagulopathy.^14^, ^15^ However, leptospirosis is known to induce hypercoagulability in some cases (as seen in our patient), with measurable increases in prothrombotic indicators like fibrinogen, thrombin‐antithrombin III complex, and prothrombin fragments.^14^, ^16^ Hemostatic dysregulation is corroborated in animal models, producing both hypercoagulable and hypocoagulable phenotypes—higher mortality was observed in the hypocoagulable subgroup.^17^

Rhabdomyolysis in this patient is also characteristic of leptospirosis. Leptospira antigens present in muscle sarcoplasm during active infection, creating an immunological target that produces myositis and elevated creatinine kinase.^2^ Resultant myoglobinuria may also contribute to renal failure, but this conjecture requires further study.^18^

Leptospirosis is underdiagnosed because physicians lack clinical suspicion, symptomatology varies, reporting systems are poor, and serological testing is usually expensive and time‐consuming.^19^ Serological testing plays an important role in the diagnosis of leptospirosis, though the WHO recommends using the modified “Faine's criteria”—a score that determines the likelihood of leptospirosis in the absence of PCR or other high‐fidelity serological testing.^20^ The utility of Faine's criteria remains in question, as one study found that using clinical signs and epidemiological exposure alone to calculate Faine's criteria only produced 40% sensitivity and 80% specificity in the diagnosis of leptospirosis while including serological data brought these values above 90%.^21^ Quantitative polymerase chain reaction (PCR) DNA amplification and microagglutination testing (MAT) are considered the gold‐standard for diagnosing leptospirosis, though enzyme‐linked immunosorbent assays (ELISA), immunodot, loop‐mediated isothermal amplification (LAMP), antigen detection, and medium‐specific culture of blood, urine, and CSF have all been investigated.^22^, ^23^, ^24^ Every year, thousands of people die worldwide due to lack of antibiotic administration and symptomatic management. According to NYC.gov, an average of 3 cases of leptospirosis are diagnosed in New York City each year, but the case rate has been increasing during the last decade with an average of 5.7 yearly cases reported over the past 3 years.^25^ One case report of urban leptospirosis in New York City showed a case of septic shock in a patient who had an infestation of rats at their residence.^26^ Other cases of leptospirosis associated with pancreatitis have been published in literature localized to Central Europe and Sri Lanka, but no such cases in North America have ever been described.^27^, ^28^ Patients typically present with jaundice, renal failure, and bleeding. Additionally, according to studies that have validated the modified Faine's criteria a constellation of meningismus, conjunctival suffusion and myalgia should increase the clinical suspicion of leptospirosis, especially in the appropriate epidemiological setting.^21^ For hospitalized patients with severe disease, treatment includes ceftriaxone 2 g intravenous daily or doxycycline 100 mg intravenous twice daily for 7 days. For outpatient management of mild disease, patients can be given oral doxycycline 100 mg twice daily for 7 days. Other antibiotics are also effective such as penicillin.^29^ Anticoagulation therapy for portal vein thrombosis includes treatment for 3‐6 months or lifelong anticoagulation if an underlying prothrombotic disorder exists.^30^ Hypercoagulability and acute pancreatitis are relatively rare, potentially leading to misdiagnosis and delay in treatment. Clinicians faced with a patient like ours should proceed with aggressive measures given higher mortality rates with interruption in treatment. This includes fluid resuscitation, early antibiotic administration, consideration of dialysis given signs of uremia, and conducting extensive laboratory testing and imaging studies to investigate the underlying etiology of the patient's symptoms. Our case illustrates the importance in creating a good differential diagnosis that accounts for the socioeconomic background and environmental exposures of patients.

## CONFLICT OF INTEREST

The authors declare that there are no conflicts of interest regarding the publication of this article.

## AUTHOR CONTRIBUTION

IA: involved in writing case presentation, abstract, collecting data and imaging, and editing. RT: involved in collecting references and major contribution to the discussion and introduction. SW: involved in contributing to abstract and discussion. MK: involved in editing and revision of the final manuscript.
